# The Prevalence of Single and Multiple Thyroid Nodules and Its Association with Metabolic Diseases in Chinese: A Cross-Sectional Study

**DOI:** 10.1155/2020/5381012

**Published:** 2020-02-14

**Authors:** Bing Zou, Li Sun, Xin Wang, Zongtao Chen

**Affiliations:** ^1^Health Management Center, Southwest Hospital, Army Military Medical University, Chongqing 400038, China; ^2^Department of Epidemiology and Biostatistics, Southwest Hospital, Army Military Medical University, Chongqing 400038, China

## Abstract

**Purpose:**

The present study aims to investigate the prevalence of single and multiple thyroid nodules and its association with metabolic diseases in subjects who participated in the heath examination in China.

**Methods:**

This is a cross-sectional study. The participants who attend the physical examination at the Health Management Center of Southwest Hospital, Army Military Medical University, between January 2014 and December 2018, were included. Thyroid nodules were diagnosed by thyroid ultrasound. Multivariable logistic regression was used to investigate the association between metabolic diseases and nodular thyroid disease.

**Results:**

A total of 9,146 subjects were included in this study; of them, 2,961 were diagnosed with thyroid nodules, with a prevalence of 32.4%. The prevalence in women was significantly higher than that in men (45.2% *vs* 26.0%; *χ*^*2*^ = 339.56, *P* < 0.001), and the prevalence was gradually increased with age (*Z* = 20.05, *P* < 0.001), and the prevalence was gradually increased with age (

**Conclusions:**

The prevalence of thyroid nodules was relatively high. Age, female gender, and diabetes are positively associated with nodular thyroid disease. High LDL cholesterolemia is more likely to be associated with multiple thyroid sarcoidosis.

## 1. Introduction

Thyroid nodules are one or more lumps made up of abnormal clusters of thyroid cells in the thyroid gland with a variety of etiologies, which can be cystic, solid, or mixed, and are the most common thyroid diseases in clinical settings [1, 2]. Thyroid nodules can be complicated by various thyroid diseases because of its insidious onset, and most of the patients are asymptomatic in the early stage [3, 4]. The prevalence of thyroid nodules among males and females was 29.49% and 33.15% in Southeast China, respectively [2]. With lifestyle and dietary changes, diabetes, hypertension, metabolic diseases such as hyperlipidemia, fatty liver, hyperuricemia, and obesity are the main chronic diseases affecting the health of residents in China [5–8]. Previous studies show certain metabolic diseases were significantly associated with thyroid diseases, but the results are not consistent [5–11]. By investigating the prevalence of thyroid nodules and its association with metabolic diseases in participants in Southwest China, we conducted a cross-sectional study based on the subjects participating in the health checkup center, to investigate whether the potential metabolic factors are associated with thyroid nodules in the different populations.

## 2. Subjects and Methods

### 2.1. Subjects

Subjects who participated in the health checkup of the Health Management Center of Southwest Hospital between January 2014 and December 2018 were included in the present study. During this period, a total of 13,773 subjects received thyroid ultrasound; 4627 of them were excluded from the study as no sufficient information were available concerning US examination results. Overall, 9,146 subjects were included in this study. This study protocol was approved by the Institutional Review Board of Southwest Hospital, Army Military Medical University (KY2019103). Informed consent was confirmed by the board.

### 2.2. Inclusion and Exclusion Criteria

The inclusion criteria included people receiving thyroid ultrasound, abdominal ultrasound examination, with complete data involving height, weight, waist circumference, hip circumference, blood pressure, fasting blood glucose, 2-hour postprandial blood sugar, four blood lipid measurements, and blood uric acid. The individuals who had a diagnosis of thyroid disease or surgery, with serious illness, taking antithyroid drugs (iodine), and the pregnant or lactating women were excluded.

### 2.3. Detection and Ultrasound Examination

#### 2.3.1. Body Measurements

The medical history and physical examination results of the subjects were collected by the professional nursing staff. The subject was placed on the health analyzer (SK-X80) after taking off the shoes. The system automatically generates the report about height, weight, and body mass index (BMI) of the subject. Blood pressure in the right arm was measured at rest (Omron electronic sphygmomanometer HBP-9021, Omron Healthcare, Kyoto, Japan). The waist circumference was measured according to the international standard. For hip circumference, the most prominent circumference of the pelvic ring was measured.

#### 2.3.2. Biochemical Indicator Test

The subject was fasted for 8 hours at night, and 10 ml of blood specimen was taken in the morning. Fasting blood glucose (FBG) and 2-hour postprandial blood glucose (2 hPG) were detected by the hexokinase method. Triglyceride (TG) content was detected by the GPO-POD method, total cholesterol (TC) was detected by the enzymatic method, low-density lipoprotein cholesterol (LDL-C) and high-density lipoprotein cholesterol (HDL-C) were detected by the direct method, and blood uric acid (UA) was detected by the uricase peroxidase method. After completing the fasting tests (such as abdominal B-ultrasound), the subjects were asked to take glucose powder orally (75 g) and the glucose tolerance was tested, and they were not allowed to drink or eat for 2 hours, after that the 2 hPG was taken on time.

#### 2.3.3. Thyroid Ultrasound Examination

The subjects took the supine position to fully expose the neck. The measurement was performed by a professional sonographer, who conducted a multisectional scanning of the thyroid gland to record in detail the location, size, shape, boundary, internal structure, echo, and blood flow of the thyroid nodules. The sonographer also carefully described the possibility of malignant nodules and the state of regional lymph nodes. Thyroid ultrasound was performed with Philips Color Super EPIQ 7 (probe frequency 7–10 MHz/50 mm).

#### 2.3.4. Abdominal Ultrasound Examination

Subjects took the supine position to fully expose their abdomen. A professional sonographer performed the examination and observed the degree of hepatic steatosis. Abdominal ultrasound was performed using Philips Color Super EPIQ 7 (probe frequency 3.5–5 MHz/50 mm).

### 2.4. Diagnosis Criteria

#### 2.4.1. The Diagnosis of Thyroid Nodule

According to the international diagnostic criteria of Thyroid Imaging Reporting and Data System (TI-RADS). In this study, TI-RADS 0 was defined as absence of thyroid nodule and TI-RADS 1–5 as presence of thyroid nodules [12].

#### 2.4.2. The Diagnosis of Multiple Thyroid Nodules

Diagnosis of multiple thyroid nodules was defined as presence of ≥2 nodules, in one or both lobes [13].

#### 2.4.3. Diagnostic Criteria for Fatty Liver

Diffuse fatty liver can be diagnosed if two of the following three items are confirmed: (1) the near-field echo of liver is diffusely enhanced and is stronger than that of the kidney. (2) The structure of intrahepatic duct is not clearly displayed. (3) The far-field echo of liver is gradually attenuated [14].

#### 2.4.4. Diagnostic Criteria for Diabetes

(1) Normal blood glucose (NGT): FBG < 6.1 mmol/L and 2 hPG < 7.8 mmol/L. (2) Impaired glucose regulation (IGR): impaired fasting blood glucose (IFG) 6.1 mmol/L ≤ FBG < 7.0 mmol/L, or impaired glucose tolerance (IGT) 7.8 ≤ 2 hPG < 11.1 mmol/L. (3) Diabetes (DM): FBG ≥ 7.0 mmol/L or 2 hPG ≥ 11.1 mmol/L [15].

#### 2.4.5. Diagnostic Criteria for Hypertension

SBP/DBP ≥140/90 mm Hg (1 mm Hg = 0.133 kPa) and/or patients having been diagnosed with hypertension and under treatment.

#### 2.4.6. Diagnostic Criteria for Metabolic Syndrome (MS)

MS can be diagnosed based on three or more of the following items: (1) centralized obesity and/or abdominal obesity : waist circumference for men ≥90 cm and for women ≥85 cm. (2) Hyperglycemia: fasting blood glucose ≥6.1 mmol/L (110 mg/dl) or blood glucose 2 hours after glucose load ≥7.8 mmol/L (140 mg/dl) or patients diagnosed with diabetes and were under treatment. (3) Hypertension: blood pressure ≥130/85 mm Hg and/or patients diagnosed with hypertension and were under treatment. (4) Fasting TG ≥1.70 mmol/L (150 mg/dl). (5) Fasting HDL-C <1.0 mmol/L (40 mg/dl) [16].

#### 2.4.7. Diagnostic Criteria for BMI

BMI = body weight (kg)/height (m^2^). Those with BMI of <18.5, 18.5–23.9, 24–27.9, and ≥28 are considered as underweight, normal weight, overweight, and obese, respectively [17].

#### 2.4.8. The Cutoff Points of Dyslipidemia

This study confirms that the reference ranges are as follows: (1) hypercholesterolemia (high TC): TC ≥ 5.7 mmol. (2) Hypertriglyceridemia (high TG): TG ≥ 1.73 mmol/L. (3) High LDL-C: LDL-C ≥ 3.1 mmol/L. (4) Low HDL-C: HDL-C < 0.9 mmol/L [18].

#### 2.4.9. Diagnostic Criteria for Hyperuricemia

For men, UA >420 umol/L and for women, UA >350 umol/L [19].

### 2.5. Statistical Analysis

Sample size and its percentage were used for describing qualitative indicators, such as gender. Chi-square test or rank-sum test was used for analysis of intergroup differences. Cochran–Armitage analysis was used to analyze the trend of thyroid nodule prevalence changes with growing age. The association between metabolic diseases and thyroid nodules was first analyzed by univariable logistic regression, and the variables with *P* < 0.05 were included in multivariable logistic regression analysis. SPSS 22.0 statistical software was used, and the significant level was defined as two-tailed *P* < 0.05.

## 3. Results

### 3.1. Basic Information and Prevalence of Thyroid Nodules of the Subjects

A total of 9,146 subjects were enrolled in the study, with 6,119 men and 3,027 women, with an average age of 46.09 ± 9.75 years (14–89 years). A total of 2961 patients were diagnosed with thyroid nodules, with prevalence of 32.4%. The prevalence in male and female were 26.0% (*N* = 1593) and 45.2% (*N* = 1368), respectively, and the prevalence in women was significantly higher than that in men (*χ*^*2*^ = 339.56, *P* < 0.001). According to age stratification, there was a significant difference in the prevalence of thyroid nodules between different age groups (*Z* = 20.05, *P* < 0.001) (Table 1), and the prevalence increased with growing age (*Z* = 20.71, *P* < 0.001). Among patients with thyroid nodules, those with single nodule accounted for 56.9% (*N* = 1685) and those with multiple nodules accounted for 43.1% (*N* = 1276).

### 3.2. Association between Metabolic Diseases and Thyroid Nodule Risk

The univariable logistic regression suggested hypertriglyceridemia (OR = 0.83; 95% CI: 0.76–0.91), IGR (OR = 1.18; 95% CI: 1.07–1.31), diabetes (OR = 1.44; 95% CI: 1.28–1.63), and hyperuricemia (OR = 0.84; 95% CI: 0.76–0.92) were all significantly associated with the development of thyroid nodule, while other factors showed no significant association (Table 2). When stratified by gender, results showed central obesity, IGR, diabetes, and metabolic syndrome were significantly associated with thyroid nodule in men, while BMI, hypertension, central obesity, hypertriglyceridemia, hypercholesterolemia, high LDL cholesterolemia, IGR, diabetes, hyperuricemia, metabolic syndrome, and fatty liver were significantly associated with it in women (Table 2).

After incorporating the age and gender and the aforementioned factors with significant association with thyroid nodule risk, multivariate logistic regression analysis indicated the female gender (OR = 2.25; 95% CI: 2.05–2.47), age (OR = 1.73; 95% CI: 1.53–1.95), and diabetes (OR = 1.24; 95% CI: 1.09–1.41) were positively associated with thyroid nodule risk in the general population. Moreover, advanced age (OR = 1.67; 95% CI: 1.43–1.95), central obesity (OR = 1.19; 95% CI: 1.06–1.34), and diabetes (OR = 1.21; 95% CI: 1.03–1.42) were positively associated with thyroid nodule risk in men, while advanced age (OR = 1.82; 95% CI: 1.49–2.23) and fatty liver (OR = 1.34; 95% CI: 1.11–1.60) were positively associated with thyroid nodule risk in women (*P* < 0.05) (Table 3).

### 3.3. Association between Metabolic Diseases and Multiple Thyroid Nodules Risk

In patients with thyroid nodules (*N* = 2961), univariable logistic regression showed that hypertension, high LDL cholesterolemia, IGR, and diabetes were significantly associated with onset of multiple thyroid nodules, while other factors suggested no association. The stratified analysis showed that BMI and hypertension were significantly associated with multiple thyroid nodules in men, while higher BMI, central obesity, hypertriglyceridemia, hypercholesterolemia, high LDL cholesterolemia, low HDL cholesterolemia, hyperuricemia, IGR, diabetes, metabolic syndrome, and fatty liver were significantly associated with multiple thyroid nodules in women (Table 4).

Multivariate logistic regression analysis showed that gender, age, and high LDL cholesterolemia were positively associated with multiple thyroid nodules risk in the general population (Table 5). When stratified by gender, results indicate that advanced age and obesity were positively associated with multiple thyroid nodules in men, and advanced age, high LDL cholesterolemia, low HDL cholesterolemia, and hyperuricemia were positively associated with multiple thyroid nodules risk in women (*P* < 0.01) (Tables 6 and 7).

## 4. Discussion

Thyroid nodule is a common disease in the general population. The prevalence of thyroid nodule diagnosis is closely related with the means of examination. The diagnosis rate by doctor's palpation is 3%–7% [20], but now it reaches as high as 50–60% in the health examination [21]. In this cross-sectional study, the prevalence of thyroid nodules in health checkup participants in Southwest China was 32.4%, which is slightly higher than that in mainland China (22.8%) [22]. There are also other countries with high prevalence of thyroid nodules (France 34.7%, Germany 23.4%, Brazil 17.0%, and Korea 13.4%) [23–27]. A number of epidemiological investigations worldwide show that the prevalence of thyroid nodules increases with age, which is consistent with the results of this study [5, 11, 28–30]. The mechanism may be that with the increase of age, the thyroid will undergo degenerative changes, leading to diffuse compensatory hyperplasia of the thyroid and eventually the nodules [31]. Therefore, thyroid ultrasound screening of the elderly should receive special attention in the future health checkup.

The results of this study show that gender is an independent risk factor for thyroid nodule, and its prevalence in women is significantly higher than that in men, which is consistent with the previous reports [22, 32, 33]. The high incidence of thyroid nodules in women is associated with increased demand for thyroid hormones during pregnancy, breastfeeding, and menstruating; estrogen can also affect the development of thyroid nodules [34].

In recent years, whether other factors contribute to the high incidence of thyroid nodules needs further investigation. Studies have shown that the incidence of thyroid nodules in patients with type 2 diabetes is significantly higher than that in healthy people [35]. It is also shown that type 2 diabetes often coexists with thyroid nodules [36, 37]. Ayturk et al. found that insulin resistance promotes the development of thyroid nodules, leading to a higher prevalence [38, 39]. Rezzonico et al. found that insulin is a growth factor for thyroid gland; therefore, high levels of insulin in the blood circulation can promote the proliferation of thyroid cells through the insulin receptor, leading to thyroid nodules [40]. Kimura showed that insulin-like growth factor (*IGF-1*) can stimulate the proliferation and differentiation of thyroid cells, which partially explains the high prevalence of thyroid nodules in diabetic patients [41]. The results of this study showed that the prevalence of thyroid nodules in the diabetic group was higher than that in the control group.

In addition, the gender stratification analysis showed that the risk factors were different between men and women. In men, diabetes and central obesity are significantly associated with the risk of thyroid nodules; in women, fatty liver has association with thyroid nodules. Studies have found that central obesity is associated with both diabetes and insulin resistance [42]. Jornayvaz et al. [10] found that insulin resistance can cause an increase in body fat, making excessive adipose tissue release nonlipidized fatty acids, and excessive fatty acids will enter the liver to induce fatty liver. The incidence of fatty liver is related to insulin resistance, which has a correlation with thyroid nodules. Therefore, in the future health checkup, thyroid ultrasound screening should be performed especially in men with diabetes and central obesity and the elderly women with fatty liver.

The associated factors for multiple thyroid nodules are rarely reported previously. To further analyze the associated factors of multiple thyroid nodules, we conducted univariate and multivariate logistic regression analysis of multiple thyroid nodules. The results showed that compared with patients with single thyroid nodule, gender, age, and high LDL cholesterolemia are significantly associated with the risk of multiple thyroid nodules in general patients. The stratified analysis showed that age and obesity were positively associated with the prevalence of multiple thyroid nodules in men, while age, high LDL cholesterolemia, low HDL cholesterolemia, and hyperuricemia were positively associated with the prevalence of multiple thyroid nodules in women.

Previous studies reported that thyroid nodule is closely related to hypertension, BMI, and metabolic syndrome [11, 43]. Our results showed that the prevalence of thyroid nodules in the patients with hypertension, BMI, and metabolic syndrome groups was higher than that in the control group, but no association was found between hypertension, BMI, metabolic syndrome, and thyroid nodules. Possible explanations include the research data are from one-time physical examination, and complete data need to be collected before enrollment. And there are differences in the research subjects (the sample size is small). Studies with large sample size will be conducted in the future to confirm the results of current study.

## 5. Conclusions

In summary, the prevalence of thyroid nodules in Southwest China is slightly higher than the average in mainland China. Age, female gender, and diabetes are positively associated with thyroid nodules risk, and high LDL cholesterolemia is more likely to associated with multiple thyroid nodules. In the future, we will conduct regular physical examinations in women and the elderly, pay attention to the screening of thyroid nodules, and identify patients with thyroid nodules early.

## Figures and Tables

**Table 1:** Basic information and prevalence of thyroid nodules of the subjects.

	Subjects with nodules (*N*, %)	Subjects without nodules (*N*, %)	*χ* ^*2*^ */Z*	*P* value
Age			20.05	<0.001
18–40	487 (20.0)	1954 (80.0)		
40–49	1179 (31.1)	2613 (68.9)		
50–59	914 (41.0)	1313 (59.0)		
60–69	318 (53.1)	281 (46.9)		
70–89	63 (72.4)	24 (27.6)		
Sex			339.56	<0.001
Female	1368 (45.2)	1659 (54.8)		
Male	1593 (26.0)	4526 (74.0)		
Total	2961 (32.4)	6185 (67.6)		

**Table 2:** Association between metabolic diseases and thyroid nodules analyzed by univariable logistic regression (*N* = 9146).

	Total				Male				Female			
	TN (*N*, %)	NTN (N, %)	OR (95% CI)	*P* value	TN (*N*, %)	NTN (*N*, %)	OR (95% CI)	*P* value	TN (*N*, %)	NTN (*N*, %)	OR (95% CI)	*P* value
BMI (kg/m^2^)												
18.5–23.9	1111 (33.0)	2253 (67.0)	1.0 (ref)	1.0	433 (24.8)	1316 (75.2)	1.0 (ref)	1.0	937 (58.0)	678 (42.0)	1.0 (ref)	1.0
24.0–27.9	1310 (32.7)	2702 (67.3)	0.98 (0.89–1.08)	0.73	811 (27.3)	2161 (72.7)	1.14 (1.00–1.31)	0.06	541 (52.0)	499 (48.0)	1.28 (1.09–1.49)	**<0.01**
≥28	540 (30.5)	1230 (69.5)	0.89 (0.79–1.01)	0.07	349 (25.0)	1049 (75.0)	1.01 (0.86–1.19)	0.89	181 (48.7)	191 (51.3)	1.46 (1.16–1.83)	**<0.01**
Hypertension												
No	2151 (32.2)	4530 (67.8)	1.0 (ref)	1.0	1100 (25.9)	3155 (74.1)	1.0 (ref)	1.0	1051 (43.3)	1375 (56.7)	1.0 (ref)	1.0
Yes	810 (32.9)	1655 (67.1)	0.97 (0.88–1.07)	0.55	493 (26.4)	1371 (73.6)	1.03 (0.91–1.17)	0.63	317 (52.7)	284 (47.3)	1.46 (1.22–1.75)	**<0.01**
Central obesity (cm)												
Male <90; female <85	1679 (31.9)	3586 (68.1)	1.0 (ref)	1.0	766 (24.5)	2359 (75.5)	1.0 (ref)	1.0	913 (42.7)	1227 (57.3)	1.0 (ref)	1.0
Male ≥90; female ≥85	1282 (33.0)	2599 (67.0)	1.05 (0.96–1.15)	0.25	827 (27.6)	2167 (72.4)	1.18 (1.05–1.32)	0.01	455 (51.3)	432 (48.7)	1.42 (1.21–1.66)	**<0.01**
High TG												
<1.73	1761 (34.2)	3395 (65.8)	1.0 (ref)	1.0	757 (26.4)	2111 (73.6)	1.0 (ref)	1.0	1004 (43.9)	1284 (56.1)	1.0 (ref)	1.0
≥1.73	1200 (30.1)	2790 (69.9)	0.83 (0.76–0.91)	**<0.01**	836 (25.7)	2415 (74.3)	0.97 (0.86–1.08)	0.55	364 (49.3)	375 (50.7)	1.24 (1.05–1.47)	**0.01**
High TC (mmol/L)												
<5.7	2185 (32.1)	4624 (67.9)	1.0 (ref)	1.0	1178 (26.2)	3321 (73.8)	1.0 (ref)	1.0	1007 (43.6)	1303 (56.4)	1.0 (ref)	1.0
≥5.7	776 (33.2)	1561 (66.8)	1.05 (0.95–1.16)	0.32	415 (25.6)	1205 (74.4)	0.97 (0.85–1.11)	0.66	361 (50.3)	356 (49.7)	1.31 (1.11–1.55)	**<0.01**
Low HDL-C												
≥0.9	2815 (32.5)	5834 (67.5)	1.0 (ref)	1.0	1471 (26.0)	4196 (74.0)	1.0 (ref)	1.0	1344 (45.1)	1638 (54.9)	1.0 (ref)	1.0
<0.9	146 (29.4)	351 (70.6)	0.86 (0.71–1.05)	0.14	122 (27.0)	330 (73.0)	1.06 (0.85–1.31)	0.63	24 (53.3)	21 (46.7)	1.39 (0.77–2.51)	0.27
High LDL-C												
<3.1	2543 (32.2)	5353 (67.8)	1.0 (ref)	1.0	1358 (26.0)	3873 (74.0)	1.0 (ref)	1.0	1185 (44.5)	1480 (55.5)	1.0 (ref)	1.0
≥3.1	418 (33.4)	832 (66.6)	1.06 (0.93–1.20)	0.39	235 (26.5)	653 (73.5)	1.03 (0.87–1.21)	0.75	183 (50.6)	179 (49.4)	1.28 (1.03–1.59)	**0.03**
DM (mmol/L)												
Normal	1567 (30.1)	3638 (69.9)	1.0 (ref)	1.0	787 (23.5)	2557 (76.5)	1.0 (ref)	1.0	780 (41.9)	1081 (58.1)	1.0 (ref)	1.0
IGR	852 (33.7)	1674 (66.3)	1.18 (1.07–1.31)	**<0.01**	467 (26.9)	1268 (73.1)	1.20 (1.05–1.37)	**0.01**	385 (48.7)	406 (51.3)	1.31 (1.11–1.55)	**<0.01**
DM	542 (38.3)	873 (61.7)	1.44 (1.28–1.63)	**<0.01**	339 (32.6)	701 (67.4)	1.57 (1.35–1.83)	**<0.01**	203 (54.1)	172 (45.9)	1.64 (1.31–2.04)	**<0.01**
HUA (*μ*mol/L)												
Male ≤420; female ≤350	2133 (33.6)	4223 (66.4)	1.0 (ref)	1.0	1012 (26.6)	2798 (73.4)	1.0 (ref)	1.0	1121 (44.0)	1425 (56.0)	1.0 (ref)	1.0
Male >420; female >350	828 (29.7)	1962 (70.3)	0.84 (0.76–0.92)	**<0.01**	581 (25.2)	1728 (74.8)	0.93 (0.83–1.05)	0.23	247 (51.4)	234 (48.6)	1.34 (1.10–1.63)	**<0.01**
MS												
No	1949 (32.3)	4092 (67.7)	1.0 (ref)	1.0	896 (25.0)	2689 (75.0)	1.0 (ref)	1.0	1053 (42.9)	1403 (57.1)	1.0 (ref)	1.0
Yes	1012 (32.6)	2093 (67.4)	1.02 (0.93–1.11)	0.76	697 (27.5)	1837 (72.5)	1.14 (1.02–1.28)	0.03	315 (55.2)	256 (44.8)	1.64 (1.37–1.97)	**<0.01**
Fatty liver												
No	1838 (32.9)	3746 (67.1)	1.0 (ref)	1.0	807 (25.4)	2371 (74.6)	1.0 (ref)	1.0	1031 (42.9)	1375 (57.1)	1.0 (ref)	1.0
Yes	1123 (31.5)	2439 (68.5)	0.94 (0.86–1.03)	0.17	786 (26.7)	2155 (73.3)	1.07 (0.96–1.20)	0.24	337 (54.3)	284 (45.7)	1.58 (1.33–1.89)	**<0.01**

Note: TN: thyroid nodules; BMI: body mass index; TG: triglyceride; TC: total cholesterol; HDL-C: high-density lipoprotein cholesterol; LDL-C: low-density lipoprotein cholesterol; IGR: sugar adjustment; DM: diabetes mellitus; HUA: hyperuricemia; MS: metabolic syndrome.

**Table 3:** Association between metabolic diseases and thyroid nodules analyzed by univariable logistic regression (*N* = 9146).

	Factors	*β*	*SE*	*Walds*	*P* value	OR	95% CI
Total	Female	0.81	0.05	283.08	**<0.01**	2.25	2.05–2.47
	Age						
	18–40				1.0	1.0 (ref)	
	41–50	0.55	0.06	75.32	**<0.01**	1.73	1.53–1.95
	51–60	0.94	0.07	187.11	**<0.01**	2.56	2.24–2.93
	61–70	1.39	0.10	193.59	**<0.01**	4.00	3.29–4.86
	>70	2.16	0.25	74.53	**<0.01**	8.67	5.31–14.16
	High TG	0.01	0.05	0.03	0.87	1.01	0.91–1.12
	Normal blood sugar				1.0	1.0 (ref)	
	IGR	0.07	0.06	1.48	0.22	1.07	0.96–1.19
	DM	0.21	0.07	10.14	**<0.01**	1.24	1.09–1.41
	HUA	0.05	0.05	0.90	0.34	1.05	0.95–1.17

Male	Age						
	18–40				1.0	1.0 (ref)	
	41–50	0.51	0.08	42.19	**<0.01**	1.67	1.43–1.95
	51–60	0.88	0.08	103.13	**<0.01**	2.42	2.04–2.87
	61–70	1.45	0.13	130.44	**<0.01**	4.28	3.33–5.49
	>70	2.38	0.34	48.23	**<0.01**	10.77	5.51–21.07
	Central obesity	0.17	0.06	8.21	**<0.01**	1.19	1.06–1.34
	Normal blood sugar				1.0	1.0 (ref)	
	IGR	0.01	0.07	0.04	0.85	1.01	0.88–1.16
	DM	0.19	0.08	5.40	**0.02**	1.21	1.03–1.42
	MS	-0.06	0.08	0.52	0.47	0.94	0.81–1.10

Female	Age						
	18–40				1.0	1.0 (ref)	
	41–50	0.60	0.10	33.79	**<0.01**	1.82	1.49–2.23
	51–60	1.03	0.11	86.10	**<0.01**	2.81	2.26–3.50
	61–70	1.33	0.16	71.87	**<0.01**	3.77	2.78–5.13
	>70	1.99	0.36	30.98	**<0.01**	7.33	3.63–14.77
	BMI						
	18.5∼23.99				1.0	1.0 (ref)	
	24∼7.9	0.08	0.10	0.66	0.42	1.08	0.90–1.30
	≥28	0.06	0.16	0.15	0.70	1.06	0.78–1.46
	Hypertension	−0.01	0.10	0.02	0.90	0.99	0.81–1.21
	Central obesity	−0.04	0.10	0.12	0.73	0.97	0.79–1.18
	High TG	−0.14	0.10	1.92	0.17	0.87	0.71–1.06
	High TC	0.01	0.11	0.01	0.94	1.01	0.81–1.26
	High LDL-C	−0.01	0.12	0.01	0.91	0.99	0.78–1.25
	Normal blood sugar				1.0	1.0 (ref)	
	IGR	0.02	0.09	0.05	0.82	1.02	0.85–1.23
	DM	0.01	0.13	0.01	0.95	1.01	0.78–1.31
	HUA	0.09	0.11	0.70	0.40	1.09	0.89–1.35
	MS	0.17	0.11	2.61	0.11	1.19	0.96–1.47
	Fatty liver	0.29	0.09	9.54	**<0.01**	1.34	1.11–1.60

Note: TG: triglyceride; TC: total cholesterol; LDL-C: low-density lipoprotein cholesterol; IGR: sugar adjustment; DM: diabetes mellitus; BMI: body mass index; HUA: hyperuricemia; MS: metabolic syndrome.

**Table 4:** Association between metabolic diseases and multiple thyroid nodules analyzed by univariable logistic regression (*N* = 2961).

	Total				Male				Female			
	STN (*N*, %)	MTN (*N*, %)	OR (95% CI)	*P* value	STN (*N*, %)	MTN (N, %)	OR (95% CI)	*P* value	STN (N, %)	MTN (*N*, %)	OR (95% CI)	*P* value
BMI (kg/m^2^)												
18.5∼23.9	654 (58.9)	457 (41.1)	1.0 (ref)	1.0	289 (66.7)	144 (33.3)	1.0 (ref)	1.0	365 (53.8)	313 (46.2)	1.0 (ref)	1.0
24∼27.9	735 (56.1)	575 (43.9)	1.12 (0.95–1.32)	0.17	502 (61.9)	309 (38.1)	1.24 (0.97–1.58)	0.09	233 (46.7)	266 (53.3)	1.33 (1.06–1.68)	**0.02**
≥28	296 (54.8)	244 (45.2)	1.18 (0.96–1.45)	0.12	204 (58.5)	145 (41.5)	1.43 (1.07–1.91)	**0.02**	92 (48.2)	99 (51.8)	1.26 (0.91–1.73)	0.17
Hypertension												
No	1253 (58.3)	898 (41.7)	1.0 (ref)	1.0	710 (64.5)	390 (35.5)	1.0 (ref)	1.0	543 (51.7)	508 (48.3)	1.0 (ref)	1.0
Yes	432 (53.3)	378 (46.7)	1.22 (1.04–1.44)	**0.02**	285 (57.8)	208 (42.2)	1.33 (1.07–1.65)	**0.01**	147 (46.4)	170 (53.6)	1.24 (0.96–1.59)	0.10
Central obesity (cm)												
Male <90; female <85	979 (58.3)	700 (41.7)	1.0 (ref)	1.0	493 (64.4)	273 (35.6)	1.0 (ref)	1.0	486 (53.2)	427 (46.8)	1.0 (ref)	1.0
Male ≥90; female ≥85	706 (55.1)	576 (44.9)	1.14 (0.99–1.32)	0.08	502 (60.7)	325 (39.3)	1.17 (0.95–1.43)	0.13	204 (44.8)	251 (55.2)	1.40 (1.12–1.76)	**<0.01**
High TG												
<1.73	997 (56.6)	764 (43.4)	1.0 (ref)	1.0	472 (62.4)	285 (37.6)	1.0 (ref)	1.0	525 (52.3)	479 (47.7)	1.0 (ref)	1.0
≥1.73	688 (57.3)	512 (42.7)	0.97 (0.84–1.13)	0.70	523 (62.6)	313 (37.4)	0.99 (0.81–1.21)	0.93	165 (45.3)	199 (54.7)	1.32 (1.04–1.68)	**0.02**
High TC (mmol/L)												
<5.7	1262 (57.8)	923 (42.2)	1.0 (ref)	1.0	730 (62.0)	448 (38.0)	1.0 (ref)	1.0	532 (52.8)	475 (47.2)	1.0 (ref)	1.0
≥5.7	423 (54.5)	353 (45.5)	1.14 (0.97–1.35)	0.12	265 (63.9)	150 (36.1)	0.92 (0.73–1.16)	0.50	158 (43.8)	203 (56.2)	1.44 (1.13–1.83)	**<0.01**
Low HDL-C												
≥0.9	1593 (56.6)	1222 (43.4)	1.0 (ref)	1.0	921 (62.6)	550 (37.4)	1.0 (ref)	1.0	672 (50.0)	672 (50.0)	1.0 (ref)	1.0
<0.9	92 (63.0)	54 (37.0)	0.77 (0.54–1.08)	0.13	74 (60.7)	48 (39.3)	1.09 (0.74–1.59)	0.67	18 (75.0)	6 (25.0)	0.33 (0.13–0.85)	**0.02**
High LDL-C												
<3.1	1477 (58.1)	1066 (41.9)	1.0 (ref)	1.0	854 (62.9)	504 (37.1)	1.0 (ref)	1.0	623 (52.6)	562 (47.4)	1.0 (ref)	1.0
≥3.1	208 (49.8)	210 (50.2)	1.40 (1.14–1.72)	**<0.01**	141 (60.0)	94 (40.0)	1.13 (0.85–1.50)	0.40	67 (36.6)	116 (63.4)	1.92 (1.39–2.65)	**<0.01**
DM (mmol/L)												
Normal	929 (59.3)	638 (40.7)	1.0 (ref)	1.0	505 (64.2)	282 (35.8)	1.0 (ref)	1.0	424 (54.4)	356 (45.6)	1.0 (ref)	1.0
IGR	464 (54.5)	388 (45.5)	1.22 (1.03–1.44)	**0.02**	290 (62.1)	177 (37.9)	1.09 (0.86–1.39)	0.46	174 (45.2)	211 (54.8)	1.44 (1.13–1.85)	**<0.01**
DM	292 (53.9)	250 (46.1)	1.25 (1.02–1.52)	**0.03**	200 (59.0)	139 (41.0)	1.25 (0.96–1.62)	0.10	92 (45.3)	111 (54.7)	1.44 (1.05–1.96)	**0.02**
HUA (*μ*mol/L)												
Male ≤420; female ≤350	1220 (57.2)	913 (42.8)	1.0 (ref)	1.0	631 (62.4)	381 (37.6)	1.0 (ref)	1.0	589 (52.5)	532 (47.5)	1.0 (ref)	1.0
Male >420; female >350	465 (56.2)	363 (43.8)	1.04 (0.89–1.23)	0.61	364 (62.7)	217 (37.3)	0.99 (0.80–1.22)	0.91	101 (40.9)	146 (59.1)	1.60 (1.21–2.12)	**<0.01**
MS												
No	1116 (57.3)	833 (42.7)	1.0 (ref)	1.0	569 (63.5)	327 (36.5)	1.0 (ref)	1.0	547 (51.9)	506 (48.1)	1.0 (ref)	1.0
Yes	569 (56.2)	443 (43.8)	1.04 (0.90–1.22)	0.59	426 (61.1)	271 (38.9)	1.11 (0.90–1.36)	0.33	143 (45.4)	172 (54.6)	1.30 (1.01–1.67)	**0.04**
Fatty liver												
No	1046 (56.9)	792 (43.1)	1.0 (ref)	1.0	506 (62.7)	301 (37.3)	1.0 (ref)	1.0	540 (52.4)	491 (47.6)	1.0 (ref)	1.0
Yes	639 (56.9)	484 (43.1)	1.00 (0.86–1.16)	1.00	489 (62.2)	297 (37.8)	1.02 (0.83–1.25)	0.84	150 (44.5)	187 (55.5)	1.37 (1.07–1.76)	**0.01**

Note: STN: single thyroid nodule; MTN: multiple thyroid nodules; BMI: body mass index; TG: triglyceride; TC: total cholesterol; HDL-C: high-density lipoprotein cholesterol; LDL-C: low-density lipoprotein cholesterol; IGR: sugar adjustment; DM: diabetes mellitus; HUA: hyperuricemia; MS: metabolic syndrome.

**Table 5:** Association between metabolic diseases and multiple thyroid nodules in general population analyzed by univariable logistic regression (*N* = 2961).

Factors	*β*	SE	Walds	*P* value	OR	95% CI
Female	0.49	0.08	41.17	**<0.01**	1.63	1.40–1.89
Age						
18–40				1.0	1.0 (ref)	
41–50	0.22	0.11	3.65	0.06	1.24	0.99–1.55
51–60	0.42	0.12	12.42	**<0.01**	1.52	1.20–1.91
61–70	0.63	0.15	17.47	**<0.01**	1.87	1.40–2.52
>70	1.09	0.28	14.75	**<0.01**	2.96	1.70–5.15
Hypertension	0.16	0.09	3.52	0.06	1.18	0.99–1.39
High LDL-C	0.30	0.11	7.79	**0.01**	1.35	1.09–1.67
Normal blood sugar				1.0	1.0 (ref)	
IGR	0.12	0.09	1.85	0.17	1.13	0.95–1.34
DM	0.11	0.11	1.06	0.30	1.12	0.91–1.38

Note: LDL-C: low-density lipoprotein cholesterol; IGR: sugar adjustment; DM: diabetes mellitus.

**Table 6:** Association between metabolic diseases and multiple thyroid nodules in male subjects analyzed by univariable logistic regression (*N* = 1593).

Factors	*β*	SE	Walds	*P* value	OR	95% CI
Age						
18–40				1.0	1.0 (ref)	
41–50	0.23	0.15	2.31	0.13	1.26	0.94–1.69
51–60	0.37	0.16	5.37	**0.02**	1.44	1.06–1.97
61–70	0.55	0.20	7.18	**0.01**	1.73	1.16–2.57
>70	0.81	0.39	4.23	**0.04**	2.25	1.04–.1.59
BMI						
18.5∼23.99				1.0	1.0 (ref)	
24∼27.9	0.24	0.13	3.53	0.06	1.27	0.99–1.62
≥28	0.44	0.15	8.47	**<0.01**	1.56	1.16–2.09
Hypertension	0.18	0.12	2.40	0.12	1.20	0.95–1.50

Note: BMI: body mass index.

**Table 7:** Association between metabolic diseases and multiple thyroid nodules in female subjects analyzed by univariable logistic regression (*N* = 1368).

Factors	*β*	SE	Walds	*P* value	OR	95% CI
Age						
18–40				1.0	1.0 (ref)	
41–50	0.23	0.17	1.84	0.18	1.26	0.90–1.77
51–60	0.44	0.18	6.06	**0.01**	1.55	1.09–2.19
61–70	0.68	0.22	9.40	**<0.01**	1.98	1.28–3.06
>70	1.32	0.42	9.98	**<0.01**	3.75	1.65–8.52
BMI						
18.5∼23.99				1.0	1.0 (ref)	
24∼7.9	0.13	0.14	0.97	0.33	1.14	0.88–1.49
≥28	−0.03	0.22	0.02	0.88	0.97	0.63–1.48
Central obesity	0.20	0.12	2.56	0.11	1.22	0.96–1.55
High TG	0.10	0.15	0.41	0.52	1.10	0.82–1.47
High TC	−0.00	0.16	0.00	0.98	1.00	0.73–1.35
High LDL-C	0.51	0.17	9.09	**<0.01**	1.67	1.20–2.32
Low HDL-C	−1.02	0.48	4.52	**0.03**	0.36	0.14–0.92
Normal blood sugar				1.0	1.0 (ref)	
IGR	0.20	0.13	2.41	0.12	1.23	0.95–1.52
DM	0.03	0.17	0.03	0.87	1.03	0.73–1.45
HUA	0.37	0.15	6.22	0.01	1.44	1.08–1.92
MS	−0.14	0.18	0.61	0.43	0.87	0.60–1.24
Fatty liver	0.03	0.16	0.40	0.84	1.03	0.76–1.41

Note: BMI: body mass index; TG: triglyceride; TC: total cholesterol; HDL-C: high-density lipoprotein cholesterol; LDL-C: low-density lipoprotein cholesterol; IGR: sugar adjustment; DM: diabetes mellitus; HUA: hyperuricemia; MS: metabolic syndrome.

## Data Availability

The data used to support the findings of this study are restricted by the Institutional Review Board of Southwest Hospital, Army Military Medical University, in order to protect patient privacy. Data are available from Southwest Hospital, Army Military Medical University, for researchers who meet the criteria for access to confidential data.

## Abbreviations

BMI: Body mass index
FBG: Fasting blood glucose
2 hPG: 2-Hour postprandial blood glucose
TG: Triglyceride
TC: Total cholesterol
LDL-C: Low-density lipoprotein cholesterol
HDL-C: High-density lipoprotein cholesterol
UA: Uric acid
TI-RADS: Thyroid imaging reporting and data system
NGT: Normal blood glucose
IGR: Impaired glucose regulation
IFG: Impaired fasting blood glucose
IGT: Impaired glucose tolerance
DM: Diabetes
MS: Metabolic syndrome.
